# Supplementary material for: Iterative improvement in the automatic modular design of robot swarms
Source: PeerJ Comput Sci. 2020 Dec 7;6:e322. doi: 10.7717/peerj-cs.322 (PMC7924708; doi:10.7717/peerj-cs.322)
Supplement: Supplemental Information 3 [file peerj-cs-06-322-s003.zip › argos3/doc/api/standalone/a00423_source.html]

ARGoS: plugins/robots/eye-bot/control\_interface/ci\_eyebot\_proximity\_sensor.h Source File


- Main Page
- Related Pages
- Namespaces
- Classes
- Files

- File List
- File Members

# plugins/robots/eye-bot/control\_interface/ci\_eyebot\_proximity\_sensor.h

Go to the documentation of this file.

```
00001 
00032 #ifndef CCI_EYEBOT_PROXIMITY_SENSOR_H
00033 #define CCI_EYEBOT_PROXIMITY_SENSOR_H
00034 
00035 namespace argos {
00036    class CCI_EyeBotProximitySensor;
00037 }
00038 
00039 #include <argos3/core/control_interface/ci_sensor.h>
00040 #include <argos3/core/utility/math/angles.h>
00041 
00042 namespace argos {
00043 
00044    class CCI_EyeBotProximitySensor : public CCI_Sensor {
00045 
00046    public:
00047 
00048       struct SReading {
00049          Real Value;
00050          CRadians Angle;
00051 
00052          SReading() :
00053             Value(0.0f) {}
00054 
00055          SReading(Real f_value,
00056                   const CRadians& c_angle) :
00057             Value(f_value),
00058             Angle(c_angle) {}
00059       };
00060 
00061       typedef std::vector<SReading> TReadings;
00062 
00063    public:
00064 
00068       CCI_EyeBotProximitySensor();
00069 
00073       virtual ~CCI_EyeBotProximitySensor() {}
00074 
00078       const TReadings& GetReadings() const;
00079 
00080 #ifdef ARGOS_WITH_LUA
00081       virtual void CreateLuaState(lua_State* pt_lua_state);
00082 
00083       virtual void ReadingsToLuaState(lua_State* pt_lua_state);
00084 #endif
00085 
00086    protected:
00087 
00088       TReadings m_tReadings;
00089 
00090    };
00091 
00092    std::ostream& operator<<(std::ostream& c_os, const CCI_EyeBotProximitySensor::SReading& s_reading);
00093    std::ostream& operator<<(std::ostream& c_os, const CCI_EyeBotProximitySensor::TReadings& t_readings);
00094 
00095 }
00096 
00097 #endif
```

---

Generated on 10 Jul 2018 for ARGoS by 
 1.6.1
